# Angiomyomatous hamartoma of subglottic tracheal wall in a 12-year-old adolescent: a case report

**DOI:** 10.1186/s13256-021-03218-1

**Published:** 2022-01-17

**Authors:** Elvin M. Mendez

**Affiliations:** Medical Director of Academics, Clinical Research and Precision Medicine, Lee Physicians Group, Lee Health, 9800 S. HealthPark Drive, Suite 200, Fort Myers, FL 33908 USA

**Keywords:** Case report, Allergic rhinitis, Asthma, Childhood hamartoma, Cough, Dysphagia, Endotracheal tumors, Hamartoma, Postnasal drip, Tracheal tumors, Vocal cord dysfunction

## Abstract

**Background:**

Allergic rhinitis is the most common allergic disease encountered in a primary care setting. Diagnosis is often made clinically based on response to empiric therapy. However, with long-term treatment failure and/or atypical disease presentation, a differential diagnosis should be considered. The following is a report of an unusual and rare presentation of a subglottic tracheal angiomyomatous hamartoma in an adolescent, treated for many years as allergic rhinoconjunctivitis and asthma.

**Case presentation:**

A 12-year-old Caucasian was referred to the allergy clinic with a lifetime history of bronchospasms and rhinoconjunctivitis symptoms, treated for many years for asthma and environmental allergies. Cough, posterior nasal drainage, self-described “choking on phlegm,” and a sensation of “a flap in the throat,”, worsened 5 months prior to the initial evaluation. Puncture skin testing for common environmental allergens was negative. Spirometry, performed due to history of chronic cough, showed blunting of the forced expiratory phase. A chest X-ray, immediately ordered to rule out possible extrapulmonary obstruction, showed bilateral bibasilar infiltrates. A noncontrast computerized tomographic scan of the chest, ordered to further elucidate X-ray findings, revealed a subglottic tracheal mass. Following a subsequent transfer and admission to a tertiary hospital center, microlaryngoscopy, bronchoscopy, and microsuspension laryngoscopy were performed to remove the tracheal mass. Pathology confirmed squamous mucosa with polypoid angiomyomatous changes and chronic inflammatory features consistent with angiomyomatous hamartoma. Surgical intervention was successful, and follow-up 1 year postoperatively revealed a healthy, asymptomatic adolescent child with normal lung function.

**Conclusions:**

Although posterior nasal drainage and cough are typical presenting symptoms in the general patient population, they may be clinically impactful as they could disguise more serious medical conditions. A detailed history and careful physical examination may provide a high index of suspicion of disease, and can help work the differential diagnosis. This case presentation is the first documentation of subglottic hamartoma reported in the pediatric literature with clinical manifestation of environmental allergy and asthma symptoms.

**Supplementary Information:**

The online version contains supplementary material available at https://doi.org/10.1186/s13256-021-03218-1.

## Background

Posterior nasal drainage (PND) is a sensation of secretions draining from the nose or paranasal sinuses into the pharynx. Patients describe a sensation of something “dripping down the throat,” rhinorrhea, and constant clearing of the throat [1]. A cough may often accompany these symptoms.

However, a description of an intermittent sensation of a flap in the throat presents a diagnostic challenge. Often described as a lump lodged in the throat, the differential diagnosis may include a globus pharyngeus or globus sensation of the throat [2, 3]. Similarly, vocal cord dysfunction (VCD) or paradoxical vocal fold movement (PVFM), might be stress- or anxiety-induced, and may also present with clinical features of asthma [4].

Masses and tumors may also be considered in the differential diagnosis. However, primary tracheal neoplasms are rare [5]. The majority of tumors are typically malignant, with fibromas, schwannomas, leiomyomas, and hamartomas the most common benign tumors [6]. Weber and Grillo analyzed 84 cases of tracheal hamartoma over 17 years and suggested that the tracheal lumen is often more than 75% compromised before any localized signs or symptoms appear [7]. Wheezing appears as the most common complaint in 56% of all patients with tracheal neoplasms. [8]

Although posterior nasal drainage and chronic cough are common presenting symptoms in allergic rhinitis, they may masquerade more serious medical diseases. Presented is an adolescent, who, despite a life-long history of asthma and allergy diagnoses, described atypical symptoms not consistent with these disorders.

## Case presentation

The allergy clinic evaluated a 12-year-old Caucasian for possible environmental allergies and a persistent cough. Clinical symptoms, present since age 1 year, consisted of nasal congestion and watery eyes, typically between January and May. Marginal relief was obtained with the use of various medications, including a variety of oral antihistamines, prescribed by a primary care physician. Cough and occasional wheezing were predominant symptoms between the ages of 2 and 3 years, unrelated to physical activity, but often triggered by upper respiratory infections. Albuterol administered via nebulizer appeared to improve lower airway symptoms.

At age 10 years, the child developed persistent posterior nasal drainage, cough, nasal congestion, and rhinorrhea, at times triggering shortness of breath. Despite environmental control measures at home, including removal of all carpeting, absence of furry animals, and presence of nonsmokers, the cough persisted. The pediatrician subsequently prescribed albuterol via a nebulizer and metered-dose inhaler.

Five months before the initial office visit, progressively worsening wet cough and croup-like symptoms developed, resulting in a prescription for oral antibiotics, corticosteroids, and eventual parenteral administration of dexamethasone by primary care provider. Initially placed on a long-acting beta-agonist/inhaled corticosteroid inhaler, medications were subsequently reduced to inhaled corticosteroid and oral leukotriene inhibitor when symptoms improved. Shortness of breath, cough, or wheezing with physical activity were denied. There was neither a history of gastroesophageal reflux (GERD) nor dysphagia. Mother noted nighttime mouth-breathing due to nasal congestion. The child, however, described sleeping upright due to the sensation of intermittent blockage, in addition to triggering an urge to cough/to clear the throat. In addition, there was an intermittent feeling of the closure of upper airways by this flap, with inspiration and expiration, and a sensation of “choking on phlegm”—the attempt to clear phlegm, at times, induced vomiting.

A review of past medical history included a full-term birth and uncomplicated spontaneous vaginal delivery. Excessive spitting up of mucus and phlegm was described shortly after birth, generating a neonatal intensive care unit (NICU) evaluation and medical clearance by neonatologists. Mother denied dysphagia or difficulty swallowing. The child had a sensitive gag reflex.

Past family history revealed biological father has a history of environmental allergies. He was routinely tested for atypical mycobacterium, due to persistent cervical lymphadenopathy, employment at a zoo a decade earlier, and exposure to monkeys. He was nonresponsive to oral antibiotic therapies. Although etiology of adenopathy remained unclear, complete resolution was noted after a course of oral corticosteroids.

Additional family history revealed a paternal aunt with asthma and breast cancer, a paternal grandmother with chronic obstructive pulmonary disease, and a paternal grandfather with colon cancer. They denied immune deficiencies or rheumatologic disorders. Aside from the father’s recent diagnosis of hypothyroidism, there was no family history of autoimmune diseases. The child had no history of drug, food, insect sting, or contact allergies.

Positive findings on the child’s initial physical examination at the allergy clinic revealed loud, audible breathing, with grunting sounds appearing to emanate from upper airways. A hyponasal voice with speech was noted. Visualization of nasal passages via otoscope revealed an S-shaped nasal septal deviation, with narrowing of left nasal passages and thick, clear mucus. Oropharyngeal examination revealed a 1+ mild tonsillar hypertrophy without exudate. Lung auscultation revealed inspiratory rhonchi on posterior lung fields, and inspiratory and expiratory rhonchi on bilateral anterior lung fields. Despite these findings, the child did not appear to be in respiratory distress.

**Fig. 1:**
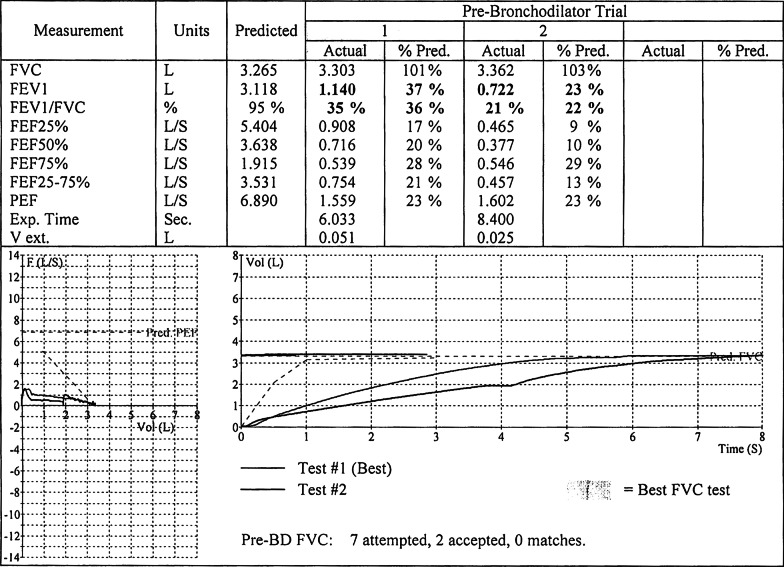
Spirometry, Pre-Surgical

Puncture skin testing to a panel of common environmental allergens was negative. Attempts to perform spirometry due to history of wheezing, lower airway complaints, cough, a flap sensation in the throat, and physical examination findings, were hampered by cough induced by the child’s respiratory efforts. The flow-volume loop revealed almost complete blunting of the forced expiratory phase (Fig. 1). Force vital capacity (FVC) was 3.943 L or 121% of predicted; Forced expiratory volume at 1 second (FEV1) was 1.119 L or 36% of predicted; FEV1/FVC ratio was at 28% or 30 % of predicted. Forced expiratory flow rate (FEF25-75%) was 0.704 L/s or 20% of the predicted value. Various disorders associated with blunting of the expiratory phase, including vocal cord dysfunction, were therefore considered.

**Fig. 2:**
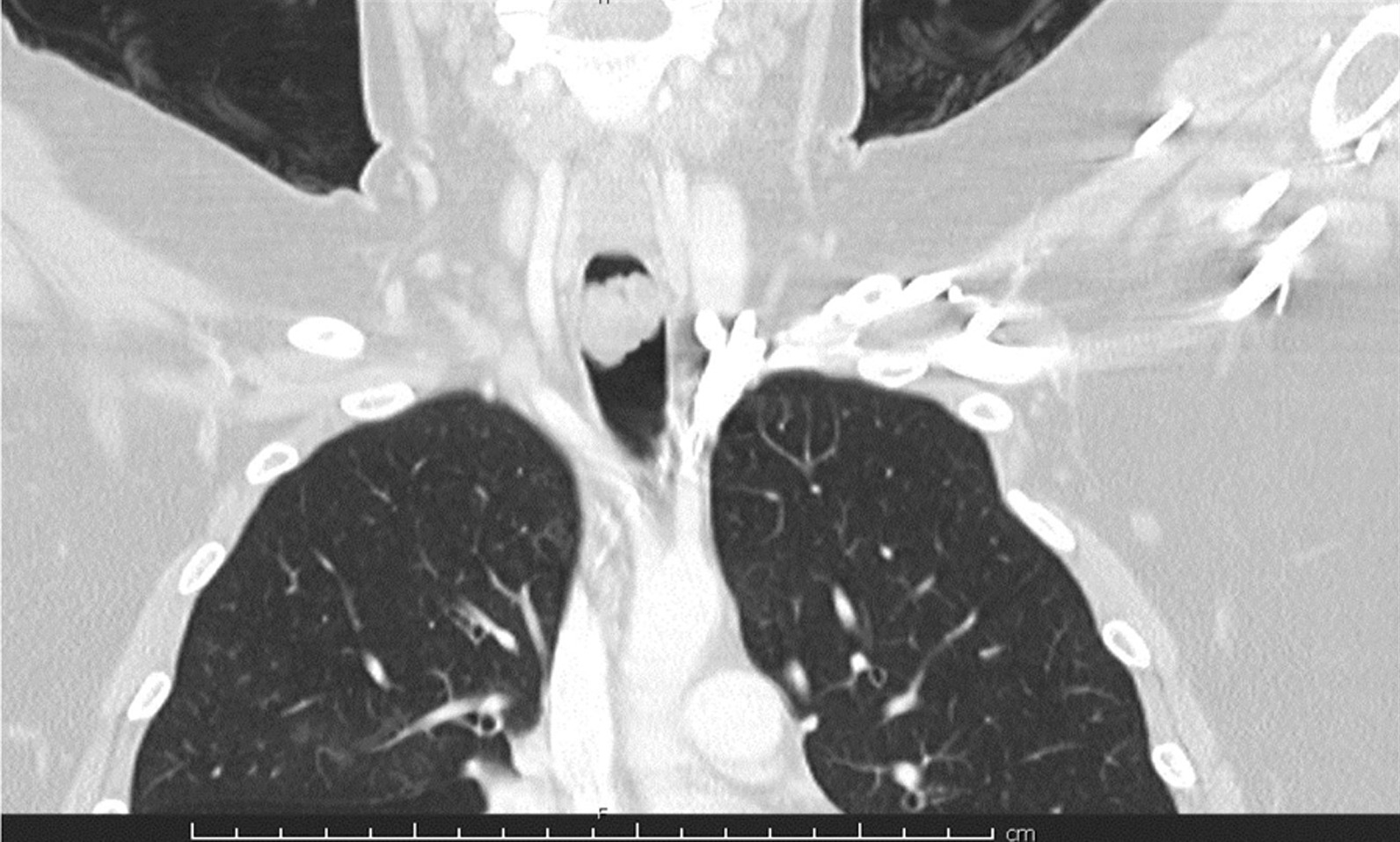
Non-Contrast Computerized Tomography of Chest, Pre-Surgical

A chest X-ray, ordered during the allergy clinic visit, revealed patchy bibasilar infiltrates and mild hyperinflation, consistent with patchy bibasilar pneumonia. Computerized tomography (CT) of the chest without contrast immediately followed, with findings including patchy air space infiltrates throughout the right lower lobe and right middle lobe, with consolidation of the left lower lobe. A prominent filling defect was noted within the proximal trachea measuring 1.3 cm in diameter (Fig. 2). Radiology further reported that the prominent filling defect within the trachea appeared attached along the posterior wall and may have reflected a large polyp. There was prominence of the trachea surrounding this, causing a possible obstructive lesion. There was patchy bibasilar infiltrates with 80% consolidation of the lower lobe.

An immediate referral was made to a local pediatric emergency department, and the patient was admitted after chest CT with contrast identified an intraluminal tracheal mass, just above the level of the clavicle or clavicular heads. The mass had a multilobular contour and measured approximately 1.5 × 1.8 × 2.6 cm. The mass also abutted both the right and left aspects of the posterior trachea and it was challenging to determine which site was the origin of the lesion. The lesion appeared to enhance slightly and nearly filled the tracheal lumen. An attempt at performing a magnetic resonance imaging (MRI) scan was unsuccessful as the child experienced a constant need to swallow when lying supine, repeatedly disrupting the quality of the image.

The patient was transferred the following day to a tertiary pediatric hospital center, where a laryngoscopy revealed almost complete subglottic and upper tracheal obstruction by a large mass emanating from the right posterolateral tracheal wall. The mass was removed, with a large amount of purulent fluid noted distal to the mass. After hospital discharge, cultures revealed moderate growth of *Staphylococcus aureus*, prompting a change from Augmentin empirical treatment of pneumonia to clindamycin. Hematoxylin and eosin (H&E) staining of tissue at 20× total magnification, as seen on Image 1 (Additional file 1), showed polypoid fragments of highly vascularized, severely edematous fibrocollagenous connective tissue lined by variable acanthotic squamous epithelial mucosa with local parakeratosis. The subepithelial area showed a heavy inflammatory infiltrate composed of neutrophils, lymphocytes, histiocytes/monocytes, plasma cells, and scattered eosinophils. H&E staining at 100× magnification on Image 2 (Additional file 2), demonstrated the subepithelial spaces were predominantly occupied by a haphazard arrangement of smooth muscle bundles and a richly vascularized stroma containing bland, mildly reactive endothelium. A focal area containing secondary inverted papillomatous-like squamous changes was noted on a single fragment of tissue. No features of malignancy were identified. No microorganisms were seen. Immunostaining of smooth muscle actin antibody via immunohistochemistry, visualized on Image 3 (Additional file 3), highlighted the haphazard arrangement of smooth muscle bundles. Pathology final diagnosis was squamous mucosa with polypoid angiomyomatous changes and chronic inflammation, features consistent with hamartoma (angiomyomatous).

**Fig. 3:**
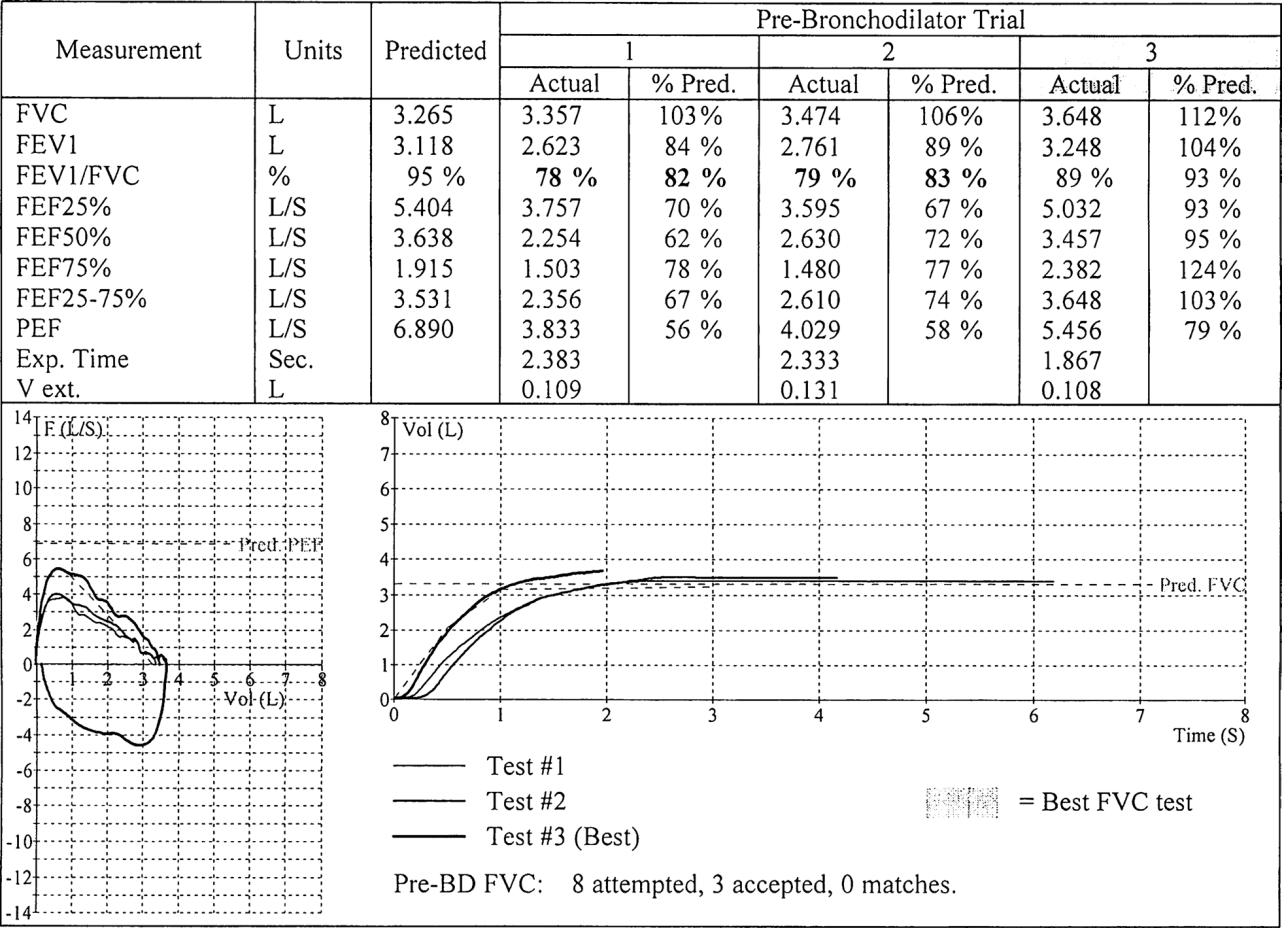
Spirometry, Post-Surgical

Follow-up with otolaryngology, shortly after hospital discharge, was uneventful, and no additional visits were necessary based on clinical findings from repeat rhinoscopy and esophagogastroduodenoscopy. Spirometry was performed on a follow-up visit to the allergy clinic, 19 days after surgical excision of the tracheal mass. Lung volumes and capacities were within the normal range (Fig. 3), prompting discontinuation of bronchodilators and an inhaled corticosteroid. Communication via a telephone call, 1-year postsurgical intervention revealed no recurrence of upper or lower respiratory tract symptoms.

## Discussion and conclusions

The differential diagnosis of posterior nasal drainage includes allergic rhinitis [9], present in 20–30% of the adult population [10] and 40% of children [11], accounts for 10–40% of visits to primary care clinics. The standard recommended diagnostic approach is a careful history, a nasal examination, and allergy tests (skin or *in vitro* test) to confirm or exclude an allergic etiology [12, 13].

General practitioners, however, may face challenges, including limited access to allergy testing, and may opt to diagnose rhinitis based on patient symptoms and response to empiric therapy. Rhinitis, whether allergic, nonallergic, infectious, or of other origin, is not possible to differentiate based solely on symptoms, especially when persistent. Negative testing to common environmental allergens made atopy or allergies an unlikely cause of this child’s initial upper airway symptoms.

Globus sensation of the throat was considered [3, 14]. A common phenomenon, this affects 12.5% of the healthy US population, and is often linked to stress and anxiety as it may present as a “lump in the throat” without the presence of dysphagia [4]. Laryngopharyngeal reflux (LPR) often mimics posterior nasal drainage and was considered [15]. Acute bronchitis was entertained as a possible diagnosis as rhonchi and cough were present on initial physical examination [16].

Extrathoracic and intrathoracic airway obstructions were considered due to frequent complaints of a flap in the throat. Although etiologies included vascular abnormalities such as vascular ring [17, 18] and laryngeal dysfunction, as seen in vocal cord dysfunction (VCD) [19], the intermittent nature of the flap described by the child throughout the interview, with worsening of symptoms when lying supine, presented a diagnostic challenge.

VCD is induced by paradoxical adduction of the vocal cords during inhalation and sometimes exhalation. Clinical presentation varies widely, from mild dyspnea to acute onset respiratory distress. As such, VCD may be misdiagnosed as asthma refractory to treatment [20]. Careful attention to the patient’s history will show a lack of response to conventional asthma therapy; patients may point to their throats when describing their respiratory symptoms and may present with noisy breathing, commonly reported by medical providers as “stridor” or “wheezing” [21]. Direct visualization of vocal cord folds via laryngoscopy confirms the diagnosis [22]. The presence of blunting, early truncation, and saw-tooth pattern of inspiratory limb of the flow-volume loop on spirometry raised a high index of suspicion; this pattern is consistent with variable extrapulmonary obstruction [23]. A chest X-ray and/or CT scan of the chest was warranted, as a mass or tumor was suspected.

Pathology confirmed diagnosis of hamartoma. Hamartomas are the most common benign lesion in adults and the second most common benign pulmonary lesion in the pediatric population [24]. Tumor-like growths, composed of mature tissues, they present at the site in which they develop. They are typically submucosal lesions that are mostly not encapsulated and have ill-defined margins. Cells may derive from primitive connective tissue such as cartilage, fat, bone, and smooth muscles. Mesenchymal hamartomas contain mesodermal cells and lack epithelial components. Glandular or epithelial hamartomas, on the other hand, refer to those containing epithelial or glandular tissue admixed with the mesodermal elements [25].

While hamartomas are the most common subtype of pulmonary tumors, they occur less frequently in the bronchi and are rare in the trachea. Primary tracheal tumors comprise < 1% of all tumors, and benign tumors are less common than malignant tracheal tumors [26].

Symptoms are gradual and persistent, dependent more on tumor size than the pathology of the tumor itself due to the slow-growing nature of these lesions. They delay diagnosis as they often mimic allergies, asthma, or chronic obstructive pulmonary disease [6]. Unlike in adults, where they tend to be asymptomatic, hamartomas can grow to be large and symptomatic in children, resulting from obstruction, presenting with symptoms of mild exertional dyspnea. There is often progression to cough, with or without expectoration. Failure to detect tracheal hamartoma may cause fatal airway obstruction, causing respiratory distress and requiring surgical excision [27, 28].

The treatment of benign primary tracheal tumors depends on the size and location of the lesion. Benign masses dictate endoscopic resection. Surgical intervention should be the initial choice in the case where a mass invading the tracheal wall is present [29].

A critical aspect of history-taking is to not only establish signs and symptoms that permit confirmation of disease, but to allow identification of those signs and symptoms that are incompatible with the diagnosis [12]. Allergic rhinitis and asthma, for example are often recognized by patients from pattern recognition of their symptoms, emphasizing the importance of eliciting patients’ observations as to what possible triggers they have identified [30]. In addition, upper and lower airway disease may appear together, prompting the need for additional diagnostic procedures. Rare disease presentations can masquerade as common symptoms, and subtle hints in a thorough history and physical examination can help work the differential diagnosis.

## Supplementary Information

**Additional file 1**. Hematoxylin and eosin (H&E) staining of polypoid mass fragment at 20x total magnification.

**Additional file 2**. Hematoxylin and eosin (H&E) staining of polypoid mass fragment at 100x total magnification.

**Additional file 3**. Immunostaining of smooth muscle actin antibody via immunohistochemistry.

## Data Availability

An evidence-based search was performed, using PubMed and Cochrane databases, for systematic reviews, case series, or case reports, using the following keywords: childhood hamartoma, hamartoma, tracheal tumors, endotracheal tumors, with inclusion criteria of children from newborn to 18 years of age, and English language. Concurrently, a search was completed using same keywords at ncbi.nlm.nih.gov, UpToDate and Elsevier web sites. 16 July 2019 and 16 May 2020. Data sharing not applicable to this article as no datasets were generated or analyzed during the current study.

## Abbreviations

PND: Postnasal drainage
VCD: Vocal cord dysfunction
PVFM: Paradoxical vocal fold movement
Exp. Time: Expiratory time
F (L/S): Flow (liter/second)
FVC: Forced vital capacity
FEV1: Forced expiratory volume at 1 second
FEV1/FVC: Forced expiratory volume/forced vital capacity
FEF25%: Forced expiratory flow rate at 25% of exhaled volume
FEF50%: Forced expiratory flow rate at 50% of exhaled volume
FEF75%: Forced expiratory flow rate at 75% of exhaled volume
FEF25–27%: Forced expiratory flow rate between 25% and 75% of the exhaled volume
L: Liter
L/S: Liter/second
PEF: Peak expiratory flow
Pre-BD: Prebronchodilator
% Pred.: Percentage of predicted
V ext.: Extrapolated volume
Vol.: Volume
