# Supplementary figures and images for: Angiomyomatous hamartoma of subglottic tracheal wall in a 12-year-old adolescent: a case report
Source: J Med Case Rep. 2022 Jan 17;16:1. doi: 10.1186/s13256-021-03218-1 (PMC8762876; doi:10.1186/s13256-021-03218-1)

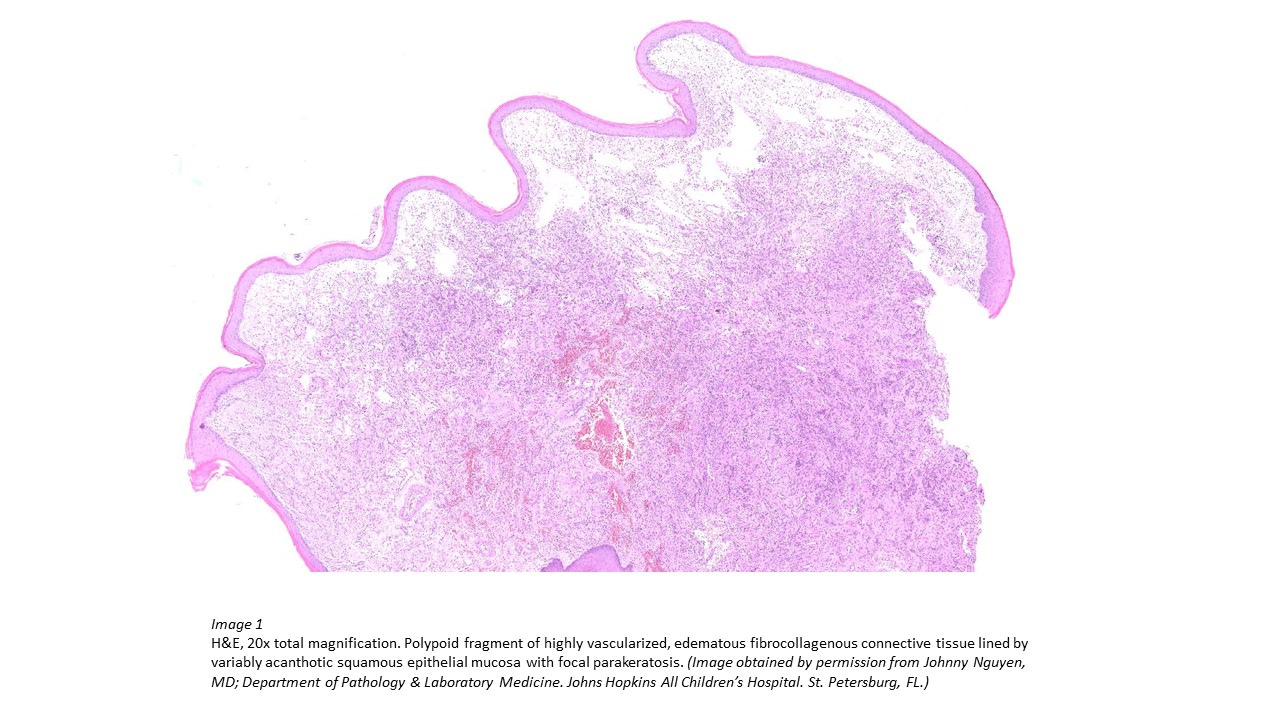

Supplement: Supplementary file 1 — Additional file 1. Hematoxylin and eosin (H&E) staining of polypoid mass fragment at 20x total magnification. [file 13256_2021_3218_MOESM1_ESM.jpg]

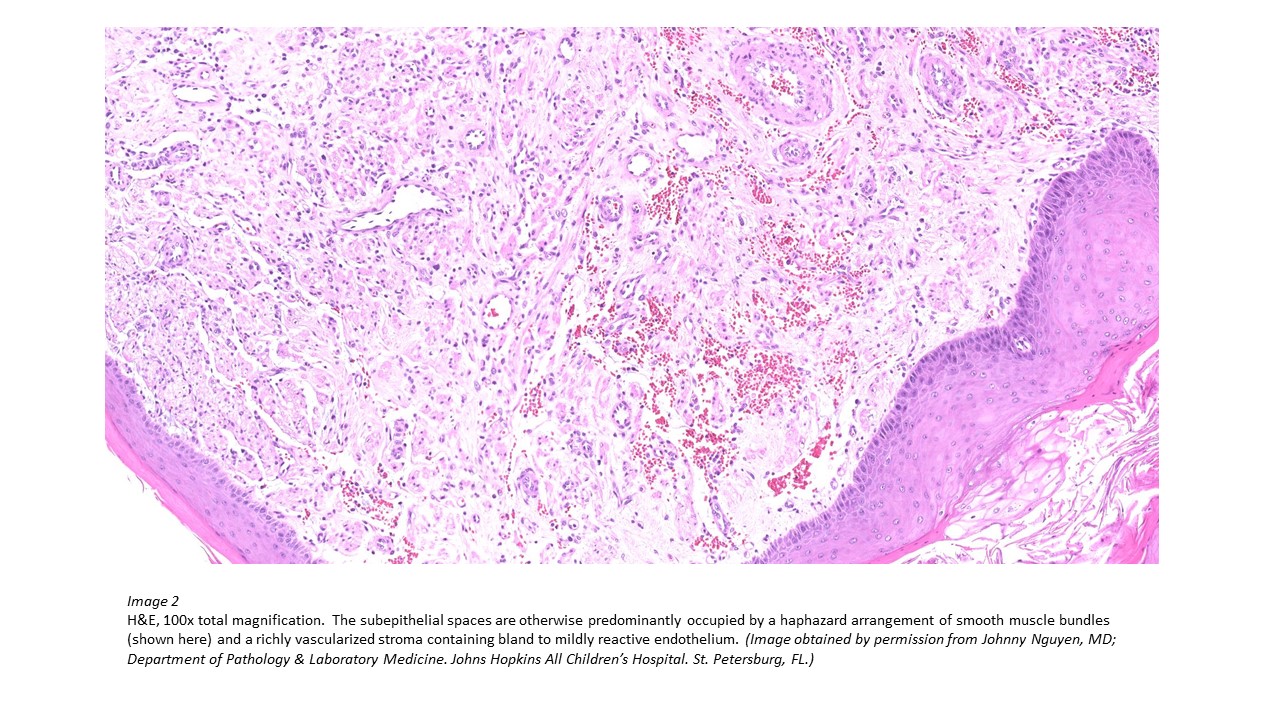

Supplement: Supplementary file 2 — Additional file 2. Hematoxylin and eosin (H&E) staining of polypoid mass fragment at 100x total magnification. [file 13256_2021_3218_MOESM2_ESM.jpg]

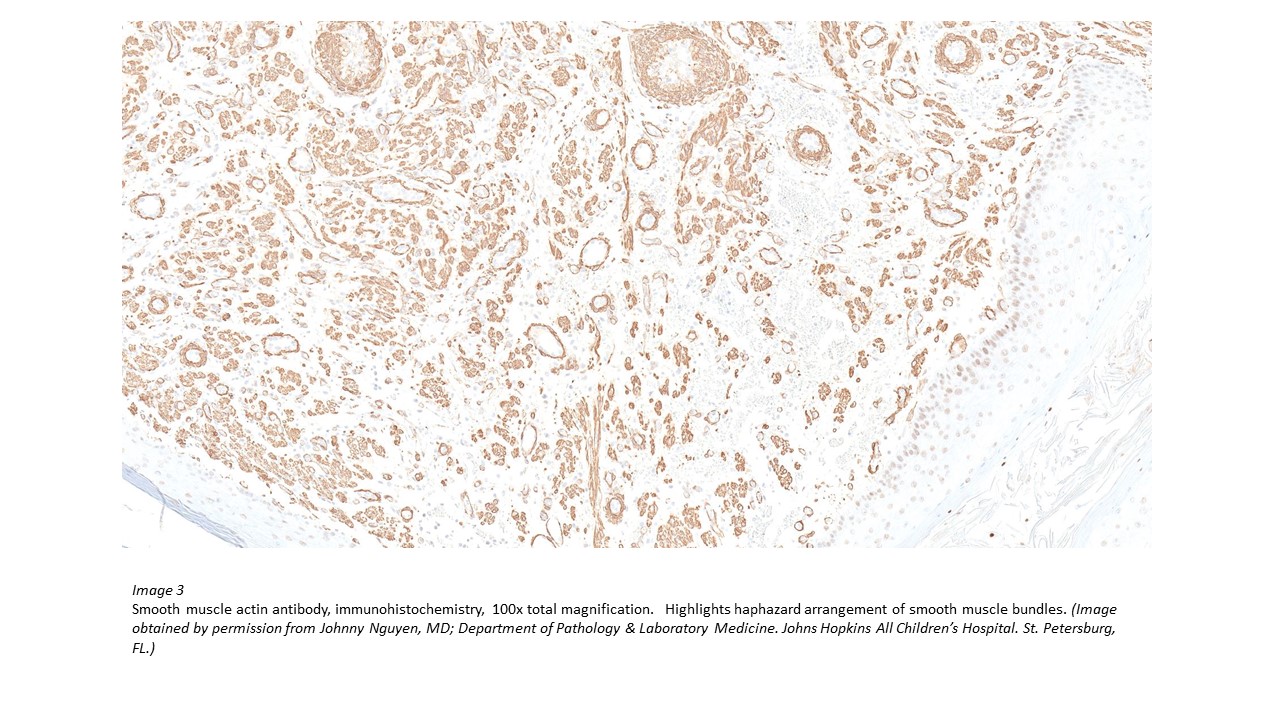

Supplement: Supplementary file 3 — Additional file 3. Immunostaining of smooth muscle actin antibody via immunohistochemistry. [file 13256_2021_3218_MOESM3_ESM.jpg]
